# Ventricular tachycardia ablation across age groups: Outcomes, trends and demographics. Insights from the National Inpatient Sample Database

**DOI:** 10.1016/j.hroo.2024.09.014

**Published:** 2024-09-27

**Authors:** Ramez Morcos, Saad Malik, Peter Hanna, Amr Barakat, Haider Al Taii, Luigi Di Biase, Jeff Winterfield, Pugazhendhi Vijayaraman, Parash Pokharel

**Affiliations:** 1 Department of Cardiology, Geisinger Heart Institute, Wilkes Barre, Pennsylvania; 2 UCLA Cardiac Arrhythmia Center, David Geffen School of Medicine, University of California, Los Angeles, Los Angeles, California; 3 Department of Cardiology, Ascension St. Vincent’s Medical Center, Jacksonville, Florida; 4 Department of Cardiology, University of Texas Medical Branch at Galveston, Galveston, Texas; 5 Department of Electrophysiology, Albert Einstein College of Medicine, Bronx, New York; 6 Division of Cardiology, Medical University of South Carolina, Charleston, South Carolina; 7 Department of Cardiology, Geisinger Medical Center, Danville, Pennsylvania

**Keywords:** Ventricular tachycardia, Ventricular tachycardia ablation, Demographic differences, Age-related outcomes, Geographic variability

## Abstract

**Background:**

Ventricular tachycardia ablation (VTA) is an important treatment option for ventricular tachycardia, with increasing use across all age groups. However, age-related differences in outcomes remain a concern.

**Objective:**

This study aimed to investigate age-related trends in VTA procedures and their associated adverse events across the United States from 2011 to 2021. The primary objective was to analyze VTA outcomes across different age groups. Secondary objectives included examining variation in VTA rate by sex and geographic region.

**Methods:**

We conducted a retrospective analysis of the National Inpatient Sample, focusing on adult patients (≥18 years of age) hospitalized with a primary diagnosis of ventricular tachycardia. Patients were divided into 3 age groups: ≤59, 60 to 79, and ≥80 years. We evaluated VTA frequency, in-hospital mortality, and complications using propensity score matching to control for confounders. Secondary analyses examined sex and geographic differences.

**Results:**

The study included 95,913 VTA procedures. The mean age of patients undergoing VTA increased over time, with a growing proportion of procedures performed in older patients. While overall adverse events did not significantly differ across age groups, specific outcomes such as mortality and hemorrhage were significantly higher in patients ≥80 years of age. In-hospital mortality was highest in the ≥80 years age group (5.1%), compared with 1.6% in the ≤59 years age group and 2.7% in the 60 to 79 years age group. Significant differences by sex and region were also observed.

**Conclusion:**

Our study demonstrates that while the overall incidence of adverse events with VTA did not significantly increase with age, specific severe outcomes, such as in-hospital mortality and hemorrhage, were more prevalent in older patients. These findings suggest that VTA can be safely performed across age groups, but careful consideration is essential for elderly patients. Future research should focus on understanding the impact of age-related physiological changes and comorbidities on VTA outcomes.

**Key Findings**

▪ There was a significant increase in ventricular tachycardia ablation (VTA) procedures among older adults over the decade, indicating an aging patient population undergoing this treatment.
▪ In propensity-matched adjusted analysis, overall adverse events were not significantly different across age groups. However, mortality and hemorrhage requiring transfusion were significantly higher in patients ≥80 years of age.
▪ Significant sex-based differences were observed in VTA procedures, with fewer females undergoing VTA compared with males throughout the decade.
▪ Geographic variations were noted, with the South consistently showing the highest proportion of VTA procedures, while the West exhibited a declining trend over the decade.
▪ These findings suggest that while VTA can be performed safely across age groups, careful consideration and individualized treatment strategies are particularly important for elderly patients.

## Introduction

Over the past decade, significant progress has been made in managing ventricular tachycardia (VT), attributable to advancements in device and medical therapy, as well as ablation techniques. Ventricular tachycardia ablation (VTA) has markedly improved our ability to control arrhythmias, especially in cases in which antiarrhythmic therapy is ineffective or undesirable. Since its pioneering application in the early 1980s, advancements in mapping and ablation techniques have enabled the treatment of various VTs previously considered unmappable. These developments have reshaped the treatment landscape, offering hope to patients with limited therapeutic options.^1^

Despite its proven efficacy, outcomes exhibit significant variability across different demographics, with age emerging as a crucial factor. Studies have shown that younger patients often achieve higher procedural success rates and experience fewer complications post-VTA. This variability can be attributed to differences in disease prevalence, physiological factors, and variations in healthcare delivery across age groups. Although VTA remains effective across all ages, older patients face a higher risk of procedural complications and generally less favorable outcomes. Furthermore, sex differences significantly influence arrhythmia treatment outcomes.^2^ Regional differences impacting VTA outcomes have been reported previously.^3^ These factors underscore the complexity of VTA outcomes and the need for comprehensive analysis.

The primary objective of this study was to examine and analyze age-related trends and adverse events (AEs) associated with VTA within the United States over the past decade. We sought to analyze how age influences VTA utilization, in-hospital mortality, and postprocedural complications. This analysis aimed to provide insights that could inform age-specific treatment strategies and improve patient care.

As a secondary objective, we present VTA trends by sex and geographic region, offering additional context to the overall landscape of VTA utilization in the United States.

## Methods

### Study design and data source

This is a retrospective analysis of data obtained from the National Inpatient Sample (NIS) from 2011 to 2021. Established by the Agency for Healthcare Research and Quality, the NIS forms a part of the Healthcare Cost and Utilization Project, representing a collaborative effort between federal entities, state agencies, and industry.^4^ The NIS stands as the largest all-payer inpatient database, offering a nationally representative dataset that is publicly available for purchase. It encompasses annual data on nearly 35 million hospitalizations from approximately 4,400 hospitals across 48 states.

### Study population

All adult hospitalizations ≥18 years of age with a primary diagnosis of VT were included in the analysis. We then identified hospitalizations that involved VTA. The International Classification of Diseases–Ninth Revision–Clinical Modification and International Classification of Diseases–Tenth Revision–Clinical Modification codes were used to determine the procedures (Supplemental Table 1). We divided the cohort into 3 age groups: ≤59 years, 60 to 79 years, and ≥80 years. The age subgroups were chosen based on clinical relevance and existing literature, providing meaningful comparisons across different stages of life.^5^ The secondary outcomes assessed included a composite of adverse outcomes and the individual components of the indexed procedure.

### Sampling and weighting

The NIS uses a stratified, systematic sampling design to create a representative sample of hospital discharges. Each year, 20% of discharges from all participating hospitals are randomly selected to form the sample. This stratified sample is designed to ensure that it is representative of all hospitalizations across the United States. The stratification is based on several hospital characteristics, including geographic region, urban/rural location, teaching status, bed size, and ownership.

### Outcomes studied and rationale

**Figure 1:**
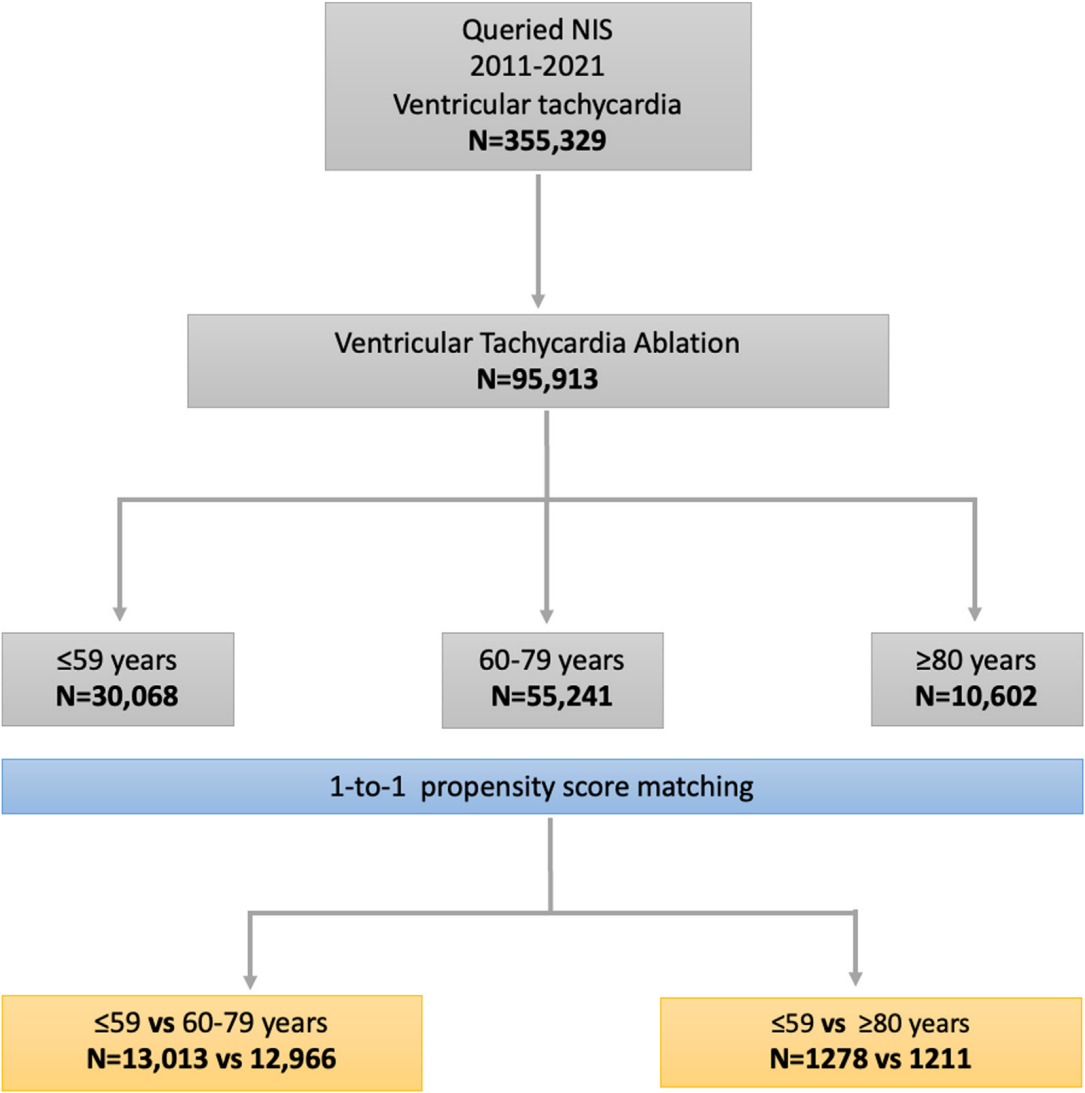
Flowchart of patient selection and propensity score matching based on age groups. NIS = National Inpatient Sample.

We used the ≤59 years age group as the control group. Comparisons were made between this control group and each of the older age groups (60–79 years and ≥80 years) (Figure 1). We focused on several adverse outcomes associated with VTA procedures. The primary outcomes studied were in-hospital mortality, defined as death occurring during the hospitalization in which the VTA was performed, and procedural complications, including myocardial infarction (MI), cardiogenic shock, pericardial effusion, transient ischemic attacks, and hemorrhage requiring blood transfusion. These outcomes were selected for their clinical relevance and potential impact on patient recovery and prognosis.

### Statistical analysis

To ensure the robustness of our analysis, we applied appropriate weights to compute national estimates. These weights account for the complex survey design of the NIS, including stratification, clustering, and unequal probabilities of selection. Descriptive statistics were utilized, including frequency, percentage, and median (interquartile range). Temporal trends were analyzed to discern the distribution of VTA over the past decade across different demographics and regions. This analysis employed chi-square tests for categorical variables, and Mann-Whitney *U* tests for continuous variables. Unadjusted analyses were conducted to evaluate the incidence of adverse outcomes associated with VTA procedures, specifically in-hospital mortality and procedural complications. Subsequently, a repeat analysis was performed after propensity score matching to control for confounding variables and reduce selection bias. The matching was conducted using a logistic regression model, adjusting for sex, race, hypertension, diabetes, peripheral vascular disease, coagulopathy, blood loss, valvular disease, chronic lung disease, chronic kidney disease, liver disease, smoking status, CHA_2_DS_2_-VASc (congestive heart failure, hypertension, age ≥75 years, diabetes mellitus, prior stroke or transient ischemic attack or thromboembolism, vascular disease, age 65–74 years, sex category) score, heart failure with preserved ejection fraction, heart failure with reduced ejection fraction (HFrEF), ischemic cardiomyopathy, and dilated cardiomyopathy. Statistical analyses were conducted using IBM SPSS Statistics for Mac, version 29.0. The significance level was set at a *P* value of <.05.

## Results

Our analysis focuses primarily on age-related trends and AEs associated with VTA. Additionally, we explore secondary trends by sex and geographic region.

### Patient characteristics

The mean age at admission from 2011 to 2021 indicates an upward trend. The mean age increased from 62.7 years in 2011 to 65.5 years in 2021, with a notable drop in 2020. The overall mean age across all years was 64.0 ± 14.2 years. The difference in mean ages between the years was statistically significant (*P <* .001). The study cohort comprised primarily White individuals (74.9%), followed by Black (13.1%), Hispanic (6.3%), Asian or Pacific Islander (2.0%), and Native American (0.4%) populations. Hypertension was prevalent in 42.8% of the cohort. Notably, the highest prevalence of hypertension was observed in the 60 to 79 years age group (46.7%; *P <* .001) compared with both the younger (<59 years) and older (≥80 years) age groups.

Diabetes affected 69.9% of patients, with a significantly higher incidence in those younger than 59 years of age (77.9%; *P <* .001). Peripheral vascular disease was reported in 8.9% of the total cohort, with a notable prevalence in the oldest group (≥80 years) at 12.3% (*P <* .001). Coagulopathy was observed in 7.2% of patients, with the highest rate in the ≥ 80 years age group (10.0%; *P <* .001). Chronic pulmonary disease affected 22.0% of the cohort, with a notably higher prevalence in the 60 to 79 years age group (25.0%; *P <* .001). Renal failure was seen in 21.3% of the cohort, significantly higher among patients ≥80 years (35.5%; *P <* .001). Smoking status showed a marked difference, with current smokers constituting 9.9% of the cohort and past smokers at 25.8%.

Atrial fibrillation was present in 23.4% of the cohort, with paroxysmal and permanent forms more prevalent in older age groups. HFrEF affected 49.4% of the cohort, with heart failure with preserved ejection fraction at 4.4%. Ischemic cardiomyopathy was noted in 40.2% of the patients and dilated cardiomyopathy in 18.4%, both showing an increase in prevalence with age (Table 1).

**Table 1:** Unadjusted baseline characteristics across age groups

Characteristic	Total (n = 95,913)		≤59 y (n = 30,068)		60–79 y (n = 55,241)		*P* value	≥80 y (n = 10,602)		*P* value
	n	%	n	%	n	%		n	%	
Female	20,454	23.9	9450	31.4	11,004	19.9	<.001	2923	27.5	<.001
Race							<.001			<.001
Asian or Pacific Islander	1623	2.0	836	2.9	786	1.5		79	0.8	
Black	10,569	13.1	5268	18.5	5301	10.1		562	5.5	
Hispanic	5114	6.3	2436	8.6	2678	5.1		414	4.1	
Native American	354	0.4	155	0.5	199	0.4		20	0.2	
White	60,658	74.9	18,652	65.5	42,006	80.0		8805	86.8	
Other	2647	3.3	1132	4.0	1514	2.9		264	2.6	
Hypertension	36,622	42.8	10,751	35.7	25,871	46.7	<.001	4836	45.4	<.001
Diabetes	59,785	69.9	23,464	77.9	36,321	65.6	<.001	3222	30.3	<.001
Peripheral vascular disease	7628	8.9	1266	4.2	6362	11.5	<.001	1304	12.3	<.001
Coagulopathy	6186	7.2	1792	5.9	4394	7.9	<.001	1063	10.0	<.001
Chronic blood loss anemia	412	0.5	110	0.4	302	0.5	.102	109	1.0	<.001
Valvular disease	665	0.8	205	0.7	461	0.8	.277	156	1.5	.001
Chronic pulmonary disease	18,804	22.0	4948	16.4	13,856	25.0	<.001	2651	24.9	<.001
Renal failure	18,246	21.3	3784	12.6	14,461	26.1	<.001	3774	35.5	<.001
Liver disease	2228	2.6	883	2.9	1345	2.4	.048	159	1.5	.001
Current smoker	8436	9.9	4064	13.5	4372	7.9	<.001	228	2.1	<.001
Past smoker	22,043	25.8	5389	17.9	16,654	30.1	<.001	3265	30.7	<.001
Atrial fibrillation							<.001			<.001
Unspecified	20,026	23.4	5477	18.2	14,549	26.3		3248	30.5	
Permanent	560	0.7	95	0.3	465	0.8		240	2.3	
Paroxysmal	11,750	13.7	2850	9.5	8900	16.1		1955	18.4	
Long-standing	210	0.2	30	0.1	180	0.3		50	0.5	
Other	2345	2.7	495	1.6	1850	3.3		550	5.2	
CHA_2_DS_2_-VASc score							<.001			<.001
0	6702	7.8	5476	18.2	1226	2.2				
1	24,474	28.6	15,559	51.6	8915	16.1				
2	26,099	30.5	6840	22.7	19,259	34.8		585	5.5	
3	17,490	20.5	1657	5.5	15,834	28.6		4502	42.3	
4	7458	8.7	528	1.8	6930	12.5		3473	32.6	
5	2518	2.9	74	0.2	2444	4.4		1487	14.0	
6	608	0.7	0	0.0	608	1.1		486	4.6	
7	152	0.2	0	0.0	152	0.3		94	0.9	
8	15	0.0	0	0.0	15	0.0		15	0.1	
HFpEF	3759	4.4	866	2.9	2892	5.2	<.001	1147	10.8	<.001
HFrEF	42,267	49.4	11,504	38.2	30,763	55.5	<.001	6269	58.9	<.001
Ischemic cardiomyopathy	34,354	40.2	7152	23.7	27,202	49.1	<.001	5309	49.9	<.001
Dilated cardiomyopathy	15,693	18.4	6452	21.4	9241	16.7	<.001	1609	15.1	<.001
Hospital bed size							.001			<.001
Small	6027	7.1	1928	6.4	4100	7.4		855	8.0	
Medium	16,005	18.7	5322	17.7	10,682	19.3		2398	22.5	
Large	63,448	74.2	22,865	75.9	40,583	73.3		7389	69.4	
Median household income							.01			<.001
0-25th percentile	20,582	24.5	7579	25.7	13,003	23.9		2126	20.4	
26th to 50th percentile (median)	20,822	24.8	7123	24.1	13,699	25.2		2616	25.0	
51st to 75th percentile	21,493	25.6	7250	24.6	14,243	26.2		2742	26.2	
76th to 100th percentile	21,021	25.0	7573	25.6	13,449	24.7		2962	28.4	
Teaching status of hospital							<.001			<.001
Rural	1236	1.4	302	1.0	934	1.7		217	2.0	
Urban nonteaching	10,139	11.9	3178	10.6	6961	12.6		1721	16.2	
Urban teaching	74,105	86.7	26,635	88.4	47,470	85.7		8704	81.8	
Elective	25,345	26	8146	27.1	14,989	27.1	<.001	2209	20.8	<.001

In compliance with the Data Use Agreement for Healthcare Cost and Utilization Project data, values <11 have been replaced with an em dash to protect patient privacy.

CHA_2_DS_2_-VASc = congestive heart failure, hypertension, age ≥75 years, diabetes mellitus, prior stroke or transient ischemic attack or thromboembolism, vascular disease, age 65–74 years, sex category; HFpEF = heart failure with preserved ejection fraction; HFrEF = heart failure with reduced ejection fraction.

### Primary outcomes

#### Age temporal trend

**Figure 2:**
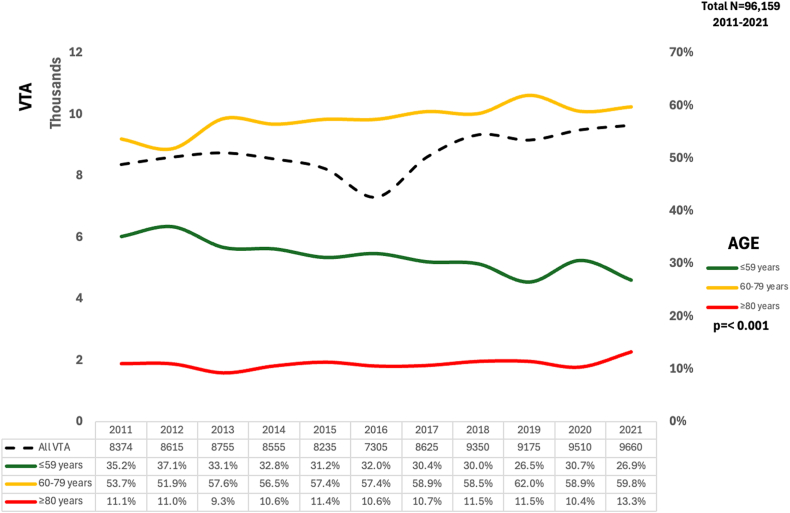
Temporal trends in ventricular tachycardia ablation (VTA) by age group. The total annual VTA procedures (dashed black line) and the percentage distribution of VTA procedures across different age groups from 2011 to 2021. The green line represents patients ≤59 years of age, the yellow line represents those 60 to 79 years of age, and the red line represents patients ≥80 years of age.

From 2011 to 2021, a total of 95,913 VTAs were performed. The annual procedural volume has shown minimal fluctuation over the last decade, with 8374 VTAs in 2011 and 9660 VTAs in 2021. Over this period, there was a gradual increase in the mean age of the patient undergoing VTA. At the start of the decade, patients younger than 59 years of age comprised 35.2% of the total cases. In 2021, this age group accounted for 26.9% of the cases. Conversely, there has been a marked increase in the proportion of procedures in patients 60 to 79 years of age, starting from 53.7% in 2011 and rising to 59.8% by 2021. Notably, the proportion of procedures performed on patients 80 years of age and older has fluctuated but showed a slightly upward trend, increasing from 11.1% in 2011 to 13.3% in 2021 (Figure 2).

#### Age-related outcomes

##### Unadjusted comparisons

The overall incidence of adverse outcomes across all age groups was 19.1%, with the highest prevalence observed in the 60 to 79 years age group (20.7%; *P <* .001). Death during hospitalization exhibited notable differences across age groups, with the oldest age group (≥80 years) experiencing a rate of 4.3% compared with 1.6% in individuals younger than 59 years of age (*P <* .001). MI complications of VTA showed an increasing trend with age, peaking at 6.1% in patients 80 years of age and older (*P =* .002). Cardiogenic shock was more prevalent in the 60 to 79 years age group (7.4%; *P =* .002), whereas the incidence in those 80 years of age and older was comparatively lower at 5.0%. Rates of pericardial effusion-tamponade were relatively stable across age groups without significant differences in prevalence. Transient ischemic attacks were more frequent in the oldest age group, with a rate of 0.4% compared with 0.1% in those younger than 59 years of age (*P =* .003). Hemorrhage requiring blood transfusion showed an age-dependent increase, being highest among those 80 years of age or older at 5.4% (*P <* .001) (Table 2).

**Table 2:** Propensity-matched baseline characteristics across age groups

Characteristics	≤59 y (n = 13,031)		60–79 y (n = 12,966)		*P* value	≤59 y (n = 1278)		≥80 y (n = 1211)		*P* value
	n	%	n	%		n	%	n	%	
Female	2861	22.0	3065	23.6	.173	307	24	369	30.1	.115
Race					.291					.964
Asian or Pacific Islander	129	1.0	178	1.4		—		—		
Black	1685	12.9	1792	13.8		318	24. 9	297	24.2	
Hispanic	714	5.5	753	5.8		99	7.8	94	7.7	
Native American	20	0.2	44	0.3		—		—		
White	10148	77.9	9806	75.6		811	63.5	781	63.7	
Other	335	2.6	393	3.0		34	2.7	39	3.2	
Hypertension	6385	49.0	6508	50.2	.451	482	37.8	750	61.2	<.001
Diabetes	3944	30.3	4112	31.7	.265	413	32.4	388	31.7	.864
Peripheral vascular disease	927	7.1	1117	8.6	.072	115	—		9.8	.717
Coagulopathy	756	5.8	935	7.2	.046	133	10.4	95	7.7	.276
Chronic blood loss anemia	25	0.2	63	0.5	.066	—		—		.991
Valvular disease	60	0.5	87	0.7	.304	—		22	1.8	.300
Chronic pulmonary disease	2774	21.3	2970	22.9	.157	292	22.9	300	24.5	.673
Renal failure	2150	16.5	2598	20.0	.002	294	23.0	371	30.3	.054
Liver disease	259	2.0	388	3.0	.021	44	3.5	34	2.8	.643
Current smoker	1921	14.7	1430	11.0	<.001	204	16	49	4.0	.000
Past smoker	2934	22.5	3142	24.2	.154	346	27	270	22.0	.177
Atrial fibrillation					<.001					<.001
Unspecified	2673	20.5	3469	26.8		257	20.1	584	47.6	
Permanent	30	0.2	75	0.6		—		—		
Paroxysmal	1405	10.8	1525	11.8		180	14.1	30	2.4	
Long-standing	20	0.2	15	0.1		—	—	—		
Other	210	1.6	340	2.6		35	0.9	—	0.0	
CHA_2_DS_2_-VASc score					.026					<.001
0	1217	9.3	1134	8.7						
1	6212	47.7	6336	48.9						
2	3895	29.9	3613	27.9		138	10.9	197	16.0	
3	1210	9.3	1110	8.6		667	52.2	425	34.7	
4	433	3.3	607	4.7		408	31.9	457	37.3	
5	64	0.5	141	1.1		64	5	114	9.3	
6	0	0.0	13	0.1		—	—	28	2.3	
7	0	0.0	—			—	—	—		
HFpEF	576	4.4	653	5.0	.298	55	4.3	83	6.8	.220
HFrEF	6210	47.7	6288	48.5	.561	716	56	383	31.3	<.001
Ischemic cardiomyopathy	5094	39.1	5511	42.5	.038	825	64	708	57.8	.133
Dilated cardiomyopathy	2580	19.8	2662	20.5	.526	268	21	326	26.6	.128
Hospital bed size					.840					.201
Small	800	6.1	843	6.5		95	7.4	50	4.1	
Medium	2410	18.5	2431	18.8		220	17.3	240	19.6	
Large	9812	75.4	9679	74.7		962	75.3	935	76.3	
Median household income					.387					.380
0 to 25th percentile	3244	25.3	3369	26.6		417	33.6	331	27.2	
26th 50th percentile	3252	25.4	2972	23.4		308	24.8	281	23.1	
51st to 75th percentile	3176	24.8	3226	25.4		285	22.9	329	27.1	
76th to 100th percentile	3132	24.5	3120	24.6		233	18.7	274	22.6	
Teaching status of hospital					.351					<.001
Rural	171	1.3	223	1.7		15	1.2	37	3.0	
Urban nonteaching	1553	11.9	1636	12.6		118	9.3	246	20.1	
Urban teaching	11,297	86.8	11,092	85.6		1144	89.5	943	76.9	
Elective	3338	25.7	3579	27.7	.097	320	25	262	21.7	<.001

In compliance with the Data Use Agreement for Healthcare Cost and Utilization Project data, values <11 have been replaced with an em dash to protect patient privacy.

CHA_2_DS_2_-VASc = congestive heart failure, hypertension, age ≥75 years, diabetes mellitus, prior stroke or transient ischemic attack or thromboembolism, vascular disease, age 65–74 years, sex category; HFpEF = heart failure with preserved ejection fraction; HFrEF = heart failure with reduced ejection fraction.

#### Propensity-matched patient characteristics

Following propensity score matching, the cohort consisted of 29,486 patients. Among them, 22.8% were females, with a slightly higher representation in the oldest age group (≥80 years, 30.1%). The racial composition primarily comprised White individuals (76.8%), followed by Black individuals (13.4%), Hispanic individuals (5.6%), Asian or Pacific Islander individuals (1.2%), and other minorities. Significant health comorbidities were identified, including hypertension (49.6%), with the highest prevalence in the ≥80 years age group (61.2%; *P <* .001), and diabetes (31.0%), which exhibited consistently distributed across the age groups. Peripheral vascular disease affected 7.9% of the cohort, and coagulopathy was present in 6.5% of patients, with notably higher incidence in the older age groups. Smoking status analysis revealed that 12.9% of patients were current smokers, with the highest rate observed in those younger than 59 years of age (14.7%). Past smoking was reported by 23.4% of the cohort. Atrial fibrillation demonstrated significant variation with age, with 23.6% of the cohort having an unspecified type of atrial fibrillation, notably increasing to 47.6% in patients 80 years of age and older. HFrEF affected 48.1% of patients. Notably, ischemic cardiomyopathy was observed in 40.8% of the cohort, with a higher prevalence among those 80 years of age and older (57.8%). Most patients received treatment in large hospital settings (75.0%), and the median household income distribution was relatively even across different percentiles. Most patients were treated in urban teaching hospitals (86.2%) (Table 3).

**Table 3:** Unadjusted outcomes across age groups

	Total (n = 95,913)			≤59 y (n = 30,068)			60–79 y (n = 55,241)			*P* value	≥80 y (n = 10,602)			*P* value
	n	%	SE (%)	n	%	SE (%)	n	%	SE (%)		n	%	SE (%)	
All adverse outcomes	16,325	19.1	0.3	4842	16.1	0.5	11,483	20.7	0.4	<.001	2155	20.2	0.9	<.001
Death during hospitalization	2222	2.6	0.1	476	1.6	0.2	1746	3.2	0.2	<.001	457	4.3	0.4	<.001
Myocardial infarction complication	4446	5.2	0.2	1318	4.4	0.3	3128	5.6	0.2	<.001	649	6.1	0.5	.002
Cardiac arrest	2540	3.0	0.1	894	3.0	0.2	1646	3.0	0.2	.986	38	2.9	0.4	.906
Cardiogenic shock	5966	7.0	0.2	1848	6.1	0.3	4118	7.4	0.3	.002	535	5.0	0.5	.060
Pericardial effusion-tamponade	2394	2.8	0.1	794	2.6	0.2	1600	2.9	0.2	.332	250	2.4	0.3	.485
Pericardial window	220	0.3	0.0	65	0.2	0.1	155	0.3	0.1	.433	25	0.2	0.1	.871
Hemorrhagic stroke	123	0.1	0.0	53	0.2	0.1	70	0.1	0.0	.402	—			.357
Transient ischemic attack	149	0.2	0.0	25	0.1	0.0	124	0.2	0.0	.031	40	0.4	0.1	.003
Ischemic stroke	272	0.3	0.0	80	0.3	0.1	193	0.3	0.1	.347	33	0.3	0.1	.737
Vascular complications	752	0.9	0.1	222	0.7	0.1	529	1.0	0.1	.149	93	0.9	0.2	.530
Hemorrhage requiring transfusion	3065	3.6	0.2	773	2.6	0.2	2292	4.1	0.2	<.001	576	5.4	0.5	<.001

In compliance with the Data Use Agreement for Healthcare Cost and Utilization Project data, values <11 have been replaced with an em dash to protect patient privacy.

#### Adjusted outcomes analysis

Overall adverse outcomes were reported as follows: 17.8% in the ≤59 years age group, 19.2% in the 60 to 79 years age group (*P =* .200), and 14.8% in the ≥80 years age group (*P =* .742), with no statistically significant difference between these groups. The incidence of death during hospitalization was 2.0% in the ≤59 years age group, 2.7% in the 60 to 79 years age group (*P =* .126), and significantly higher at 5.1% in the ≥80 years age group (*P =* .009). Hemorrhage requiring transfusion occurred in 2.7% of the ≤59 years age group, 3.8% of the 60 to 79 years age group (*P =* .028), and 5.4% of the ≥80 years age group (*P =* .026), showing significant differences in the older age groups. MI complications of VTA rates were 5.4% in the ≤59 years age group, 5.5% in the 60 to 79 years age group (*P =* .823), and 2.0% in the ≥80 years age group (*P =* .011). There was no significant difference in the incidence of cardiac arrest and cardiogenic shock across the age groups. Similarly, pericardial effusion, tamponade, and pericardial window procedures did not significantly vary across age groups. Ischemic conditions and vascular complications remained consistent across all age groups (Table 4).

**Table 4:** Adjusted outcomes across age groups

	≤59 y (n = 13,031)			60–79 y (n = 12,966)			*P* value	≤59 years (n = 1278)			≥80 years (n = 1211)			*P* value
	n	%	SE (%)	n	%	SE (%)		n	%	SE (%)	n	%	SE (%)	
All adverse outcomes	2316	17.8	0.8	2489	19.2	0.8	.200	202	15.8	2.1	182	14.8	2.2	.742
Death during hospitalization	266	2.0	0.3	350	2.7	0.3	.126	14	1.1	0.6	63	5.1	1.3	.009
Myocardial infarction complication	700	5.4	0.5	715	5.5	0.5	.823	83	5.6	1.4	25	2.0	0.9	.011
Cardiac arrest	405	3.1	0.3	437	3.4	0.3	.583	14	1.1	0.5	37	3.0	0.9	.065
Cardiogenic shock	864	6.6	0.5	828	6.4	0.5	.726	73	5.7	1.4	35	2.8	0.9	.062
Pericardial effusion-tamponade	332	2.5	0.3	391	3.0	0.4	.333	—		0.4	—			.511
Pericardial window	15	0.1	0.1	50	0.4	0.1	.057	—		0.4				
Hemorrhagic stroke	28	0.2	0.1	—			.184	18	1.4	0.7	—			.178
Transient ischemic attack	—			—			.997							
Ischemic stroke	50	0.4	0.1	58	0.4	0.1	.716	—			18	1.4	0.7	.208
Vascular complications	143	1.1	0.2	141	1.1	0.2	.980	19	1.5	0.6	13	1.1	0.5	.599
Hemorrhage requiring transfusion	351	2.7	0.3	494	3.8	0.4	.028	24	1.9	0.8	67	5.4	1.2	.026

In compliance with the Data Use Agreement for Healthcare Cost and Utilization Project data, values <11 have been replaced with an em dash to protect patient privacy.

### Secondary outcomes

#### Sex temporal trend

**Figure 3:**
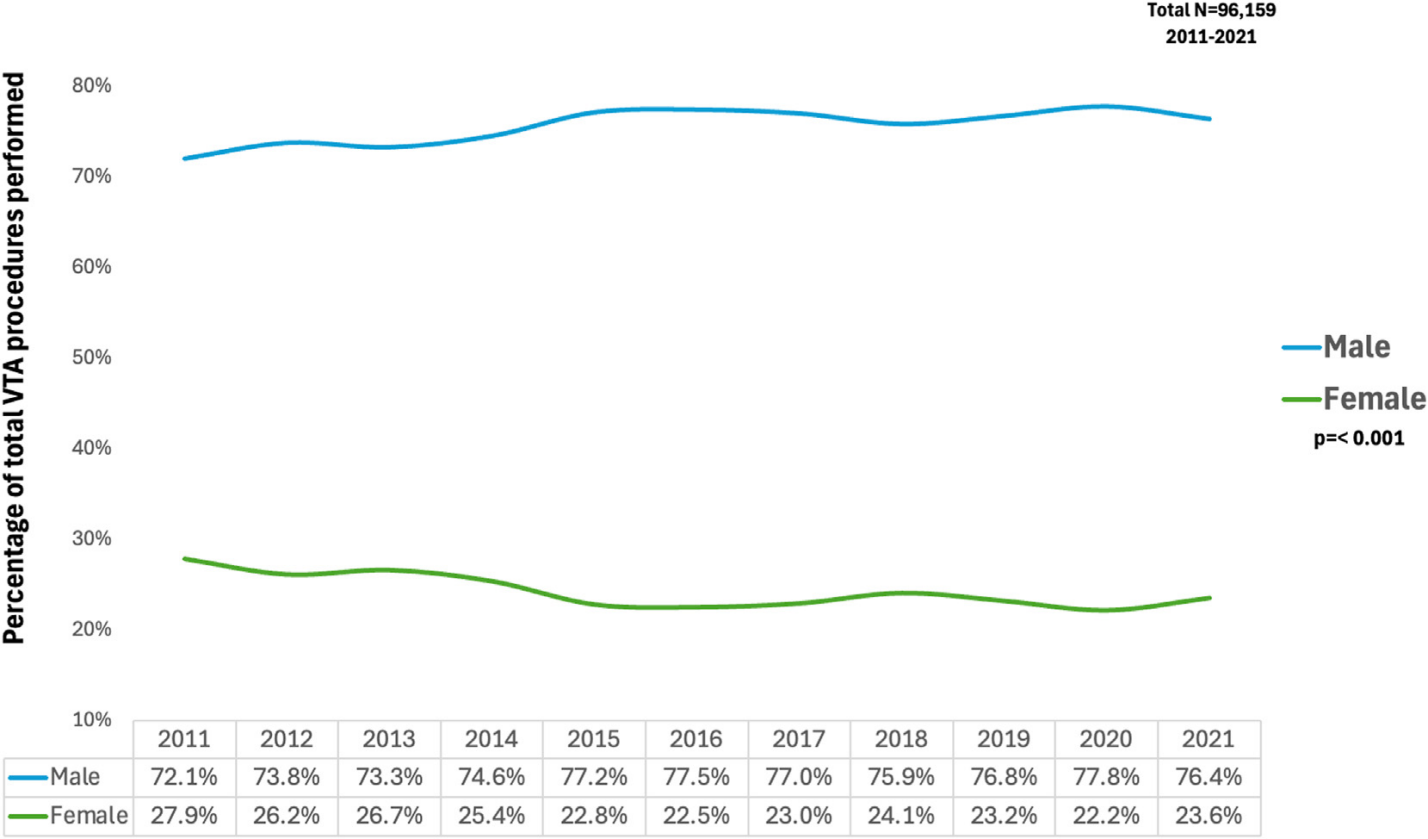
Temporal trends in the percentage of total ventricular tachycardia ablation (VTA) procedures performed by sex from 2011 to 2021. The *P* value for the distribution of procedures between males and females is <.001.

From 2011 to 2021, the portion of VTAs performed on males consistently exceeded those on females (Figure 3). In 2011, 72.1% of VTAs were performed on males, with females comprising 27.9%. This sex gap reached its widest point in 2015, with 77.2% of the VTA performed on males and only 22.8% on females. While the trend has slightly reversed over the subsequent years, males still comprised most of the VTA patient population. By 2021, 76.4% of VTA was still carried out on males, while females accounted for 23.6%. Throughout the decade, there has been a statistically significant difference in the distribution of VTA between males and females (*P* < .001).

#### Geographical distribution temporal trend

**FIGURE 4:**
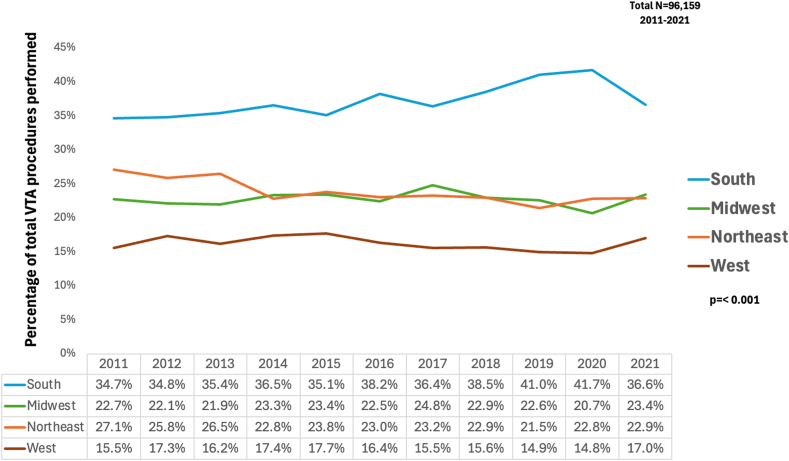
Temporal trends in the percentage of total ventricular tachycardia ablation (VTA) procedures performed across different U.S. regions from 2011 to 2021. The *P* value for the geographical distribution of procedures is <.001.

The distribution of VTAs across different U.S. regions shows significant geographical variations from 2011 to 2021. The South has consistently exhibited the highest proportion of VTAs, reaching 41.7% in 2020 before slightly decreasing to 36.6% in 2021. In contrast, the Midwest and Northeast have shown more stable percentages over the decade. The Midwest experienced a minor peak in 2017 at 24.8%, and the Northeast maintained around 23% throughout most of the period. Conversely, the West region had the lowest percentage of VTAs, with numbers declining over the decade. In 2020, it reached its lowest point at 14.8%, followed by a slight increase to 17.0% in 2021. These geographical variations were statistically significant (*P* < .001) (Figure 4).

## Discussion

We retrospectively examined a national database of patients who underwent VTA during hospitalization using data from NIS. Our primary objective was to analyze age-related trends and AEs associated with VTA. Additionally, we explored trends by sex and geographic region across the United States.

We employed rigorous methodological frameworks, including unadjusted analyses, propensity score matching, and adjusted outcomes analysis. We used the ≤59 years age group as the control group, comparing it with 2 older age groups (60–79 years and ≥80 years), rather than performing simultaneous comparisons among all 3 age groups. This method was chosen to emphasize the differences between younger patients and older cohorts, providing a clearer understanding of age-specific trends and outcomes.

Our data show an increasing trend in VTA procedures in older age groups. This trend may stem from advancements in medical care and better management of comorbid conditions, making older patients viable candidates for VTA. Additionally, increased life expectancy could contribute to a larger elderly population requiring such interventions. However, our study does not provide evidence for the underlying causes of these trends.

Unadjusted comparisons indicated differences in outcomes such as MI complications of VTA, cardiogenic shock, and hemorrhage requiring blood transfusion across different age groups. The incidence of MI complications of VTA was higher in older age groups, suggesting an age-related increase in risk, likely due to physiological changes and a higher burden of comorbidities with age.

After propensity score matching and adjusted outcome analyses, we found that overall rates of adverse outcomes did not differ significantly with age: 17.8% in the ≤59 years age group vs 19.2% in the 60 to 79 years age group (*P =* .200) and 15.8% in the ≤59 years age group vs 14.8% in the ≥80 years age group (*P =* .742). However, specific severe outcomes were significantly higher in older cohorts. Death during hospitalization was significantly higher in the ≥80 years age group (5.1%) compared with the ≤59 years age group (1.1%; *P =* .009). Similarly, hemorrhage requiring transfusion was significantly higher in the ≥80 years age group (5.4%) compared with the ≤59 years age group (1.9%; *P =* .026). These findings underscore the importance of considering these specific risks when assessing older patients for VTA, as the adjusted analysis shows significant differences in mortality and hemorrhage despite overall AEs not differing significantly.

Interestingly, we observed a significantly lower rate of MI in older patients (≥80 years) compared with younger groups after adjusted analysis. This finding was unexpected and may be explained by survivor bias, in which older patients undergoing VT ablation represent a selected group of healthier individuals, or by treatment selection bias, with physicians potentially being more selective in recommending VT ablation for older patients with lower cardiovascular risk profiles. Additionally, older patients might be more likely to be on cardioprotective medications. However, we cannot exclude the possibility of unaccounted confounders or limitations in our study design. Further research is needed to confirm and explain this unexpected association.

Our study provides important insights into the relationship between age and outcomes in VTA. While previous literature^6^^,^^7^ has placed significant emphasis on age as a predictor of outcomes, our findings suggest a more complex picture. In our unadjusted analysis, we observed results consistent with prior studies, showing an apparent association between advanced age and increased complications. However, our adjusted analysis revealed a different story. After controlling for confounding factors, we found no statistically significant differences in overall outcomes among different age groups undergoing VT ablation.

This discrepancy between unadjusted and adjusted results highlights the importance of considering multiple factors when assessing risk in VT ablation procedures. While age remains an important clinical consideration, our study suggests that it may not be as determinative of outcomes as previously thought when other relevant factors are taken into account. These findings challenge the heavy weighting of age in current risk stratification tools and underscore the need for a more comprehensive approach to patient assessment and selection for VT ablation. This insight may help inform clinical decision making and potentially expand access to VT ablation for older patients who might otherwise be deemed too high risk based solely on age criteria.

Our analysis also revealed significant sex differences in VTAs performed over the past decade. Notably, fewer females underwent VTAs than males, aligning with findings from Olarte and colleagues^8^ on sex-specific differences in VT outcomes and reflecting the higher prevalence of VT in men and diagnostic and treatment biases.

We observed regional variations in the number of VTA procedures, with the South consistently showing the highest numbers. The Midwest and Northeast showed stable trends, while the West experienced a decline. This pattern could indicate regional differences in healthcare infrastructure, referral patterns, or disease prevalence. However, our data do not explain these regional differences, and further research is needed to understand the underlying factors contributing to these variations.

### Limitations

This study's retrospective analysis of NIS data has several limitations. The use of secondary data may have introduced surrogate information bias and made it challenging to account for all confounding variables. The NIS database lacks detailed clinical information on VT severity, recurrence, and imaging, limiting our understanding of the ablations' context. Additionally, using International Classification of Diseases codes for condition and procedure identification may have led to misclassification bias.

Despite employing propensity score matching to balance baseline characteristics, the potential for residual confounding remains, which could explain some unexpected findings. Future studies should address these limitations, further investigate unexpected results, and evaluate the economic impact of VTAs to provide a more comprehensive understanding of their benefits and drawbacks.

## Conclusion

Our study provides critical insights into the age-related trends and AEs associated with VTA over the past decade, reflecting changing demographics and treatment patterns. Despite the aging patient populations, the overall incidence of adverse events in this analysis did not significantly escalate with age, suggesting that VTA can be performed safely across different age groups. However, specific severe outcomes, such as in-hospital mortality and hemorrhage requiring transfusion, were more prevalent among older patients. These results underscore the importance of careful consideration and individualized treatment strategies, particularly for elderly patients undergoing VTA. To optimize the safety and efficacy of VTA for all patients, future research should aim to understand better physiological changes with age, comorbidities, and their impact on VTA outcomes.
