# Supplementary material for: Ventricular tachycardia ablation across age groups: Outcomes, trends and demographics. Insights from the National Inpatient Sample Database
Source: Heart Rhythm O2. 2024 Sep 27;5(12):900–9. doi: 10.1016/j.hroo.2024.09.014 (PMC11721718; doi:10.1016/j.hroo.2024.09.014)
Supplement: Table S1 [file mmc1.docx]

| **Condition** | **ICD-9** | **ICD-10** |
| --- | --- | --- |
| Atrial fibrillation | 42731 | I4891, I4821, I480, I4811, I4819, |
| HFpEF | 428.30-428.33 | I50.30-I50.33 |
| HFrEF | 402.01, 402.11, 402.91, 404.01, 404.03, 404.11, 404.13, 404.91, 404.93, 428.1, 428.20-428.23 | I11.0, I13.0, I13.2, I50.1, I50.20-I50.23, I50.40-I50.43, I50.80, I50.81 with its subgroups, I50.82, I50.83, I50.84, I50.89 |
| Ischemic Cardiomyopathy | 4148, 412 | I25.5, I25.89, I25.9, I25.2 |
| Dilated Cardiomyopathy | 4254 | I420 |
| Myocardial infarction | 410.01, 410.02, 410.1, 410.11, 410.12, 410.2, 410.21, 410.22, 410.3, 410.31, 410.32, 410.4, 410.41, 410.42, 410.5, 410.51, 410.52, 410.6, 410.61, 410.62, 410.7, 410.71, 410.72, 410.8, 410.81, 410.82, 410.9, 410.91, 410.92 | I210, I2102, I2109, I2111, I2119, I2121, I2129, I213, I214, I219, I21A1, I21A9, I220, I221, I222, I228, I229 |
| Cardiac arrest | 427.5 | I469, I462, I468 |
| Cardiogenic shock | 78551 | R570 |
| Pericardial effusion | 4239 | I31.4, I31.2, I31.3 |
| Tamponade | 423.3 | I31.4 |
| Hemorrhagic stroke | 430, 431, 432 | I610-I619 |
| Ischemic stroke | 434.01, 434.11, 434.91, 433.01, 433.11, 433.21, 433.31, 433.81, 433.91, 434.01, 434.11, 434.91, 436 | I630 |
| TIAs | 435.0, 435.1, 435.2, 435.3, 435.8, 435.9 | G450-G459 |
| Vascular complication | 9040, 9041, 9042, 9044, 90440, 90441, 9047, 9048, 9049, 9982, 9992, 9972, 9977, 99779 | S7510, S75101, S75102, S75109, S7511, S75111, S75112, S75119, S7512, S75121, S75122, S75129, S7519, S75191, S75192, S75199, S35514, S35515, S35516, S3510, S3511, S3512, S3519, S2540, S25401, S25402, S25409, S2541, S25411, S25412, S25419, S2542, S25421, S25422, S25429, S2549, S25491, S25492, S25499, 9040, 9041, 9042, 9044, 90441, 9047, 9048, 9049, 9982​ |
| Hemorrhage requiring red blood cell transfusion | 9900, 9904 | 30233N0, 30233N1, 30233P0, 30233P1, 30240N0, 30240N1, 30240P0, 30240P1, 30243N0, 30243N1, 30243P0, 30243P1, 30250N0, 30250N1, 30250P0, 30250P1, 30253H0, 30253H1, 30253N0, 30253N1, 30253P0, 30253P1, 30260N0, 30260N1, 30260P0, 30260P1, 30263N0, 30263N1, 30263P0, 30263P1 |
